# Supplementary material for: Several explorations on how to construct an early warning system for local government debt risk in China
Source: PLoS One. 2022 Feb 8;17(2):e0263391. doi: 10.1371/journal.pone.0263391 (PMC8824348; doi:10.1371/journal.pone.0263391)
Supplement: S1 Appendix — (DOCX) [file pone.0263391.s001.docx]

**Appendix A. Individual risk index of local government debt.**

|  | 2010 | 2011 | 2012 | 2013 | 2014 | 2015 | 2016 | 2017 | 2018 |
| --- | --- | --- | --- | --- | --- | --- | --- | --- | --- |
| Anhui | 0.5414 | 0.5338 | 0.6352 | 0.6352 | 0.6754 | 1.0515 | 0.9673 | 1.1501 | 1.0219 |
| Beijing | 0.3468 | 0.4004 | 0.4406 | 0.4406 | 0.6029 | 1.0075 | 1.0198 | 0.8586 | 0.9242 |
| Chongqing | 0.5414 | 0.5414 | 0.6754 | 0.7156 | 0.9232 | 1.0075 | 1.3396 | 1.2711 | 0.9458 |
| Fujian | 0.4476 | 0.4476 | 0.5816 | 0.5414 | 0.6352 | 1.0477 | 1.2186 | 1.1099 | 0.8654 |
| Gansu | 0.5414 | 0.6352 | 0.7376 | 0.6352 | 0.7562 | 0.9868 | 1.0452 | 1.0452 | 1.0414 |
| Guangdong | 0.3118 | 0.3468 | 0.3066 | 0.4406 | 0.4004 | 0.6218 | 0.8649 | 0.7964 | 0.7562 |
| Guangxi | 0.5414 | 0.5816 | 0.5414 | 0.6352 | 0.8547 | 1.0477 | 1.1501 | 1.1941 | 1.1539 |
| Guizhou | 0.5414 | 0.4936 | 0.5414 | 0.6352 | 0.8547 | 1.0138 | 1.2774 | 1.2711 | 1.1831 |
| Hainan | 0.4586 | 0.5816 | 0.3758 | 0.5414 | 0.5414 | 0.6792 | 0.9453 | 0.8649 | 1.1285 |
| Hebei | 0.4476 | 0.5414 | 0.5414 | 0.5414 | 0.6792 | 0.8683 | 0.8649 | 0.9026 | 0.9868 |
| Heilongjiang | 0.5816 | 0.5414 | 0.5414 | 0.5854 | 0.7194 | 0.8806 | 1.0917 | 0.9428 | 1.049 |
| Henan | 0.4476 | 0.5414 | 0.5414 | 0.5414 | 0.6352 | 0.8281 | 1.0477 | 1.1099 | 0.9534 |
| Hubei | 0.5414 | 0.5414 | 0.5414 | 0.6352 | 0.8145 | 0.8865 | 1.0075 | 0.8988 | 0.9936 |
| Hunan | 0.5414 | 0.4936 | 0.6352 | 0.6352 | 0.8145 | 1.0515 | 1.2372 | 1.2711 | 1.1624 |
| Inner Mongolia | 0.5414 | 0.5816 | 0.5816 | 0.5854 | 0.6792 | 0.9491 | 1.2004 | 1.1357 | 1.005 |
| Jiangsu | 0.4004 | 0.4406 | 0.4004 | 0.6735 | 0.7822 | 0.9752 | 1.1178 | 1.0091 | 1.2309 |
| Jiangxi | 0.5414 | 0.5414 | 0.5414 | 0.6352 | 0.6352 | 0.9089 | 1.0477 | 1.0477 | 1.1099 |
| Jilin | 0.5414 | 0.5414 | 0.5414 | 0.5414 | 0.7194 | 0.9893 | 1.0917 | 1.0917 | 1.049 |
| Liaoning | 0.4476 | 0.4476 | 0.5414 | 0.5816 | 0.8585 | 1.0176 | 1.2664 | 1.2347 | 1.126 |
| Ningxia | 0.6352 | 0.4458 | 0.416 | 0.5414 | 0.6792 | 0.9491 | 1.0477 | 1.0075 | 1.1137 |
| Qinghai | 0.7156 | 0.5874 | 0.7778 | 0.7376 | 0.8586 | 1.0854 | 1.3151 | 1.2347 | 1.126 |
| Shananxi | 0.5414 | 0.5414 | 0.6218 | 0.6754 | 0.6352 | 1.0515 | 1.1501 | 1.1099 | 0.961 |
| Shandong | 0.4528 | 0.4476 | 0.4878 | 0.5816 | 0.7207 | 0.8281 | 1.0138 | 1.0515 | 0.9868 |
| Shanghai | 0.3066 | 0.3066 | 0.3506 | 0.4004 | 0.4004 | 0.4846 | 0.7239 | 0.6478 | 0.7008 |
| Shanxi | 0.4878 | 0.5414 | 0.5414 | 0.5854 | 0.7647 | 0.7596 | 0.9893 | 0.8108 | 0.9026 |
| Sichuan | 0.5414 | 0.4936 | 0.5414 | 0.6352 | 0.6352 | 1.0515 | 1.0477 | 1.1099 | 0.9534 |
| Tianjin | 0.5414 | 0.5414 | 0.4004 | 0.7841 | 1.0442 | 1.0515 | 1.3798 | 1.126 | 1.2787 |
| Xinjiang | 0.5414 | 0.6218 | 0.5414 | 0.6352 | 0.8145 | 1.0917 | 1.0917 | 1.0697 | 0.9056 |
| Yunnan | 0.5414 | 0.5338 | 0.5816 | 0.6352 | 0.6754 | 1.0113 | 1.2711 | 1.2711 | 1.0266 |
| Zhejiang | 0.3066 | 0.4004 | 0.4004 | 0.6199 | 0.7577 | 1.0578 | 1.0760 | 0.9673 | 0.9597 |

**Appendix B. Contagion risk index of local government debt.**

|  | 2010 | 2011 | 2012 | 2013 | 2014 | 2015 | 2016 | 2017 | 2018 |
| --- | --- | --- | --- | --- | --- | --- | --- | --- | --- |
| Anhui | 1.7148 | 1.1846 | 1.7148 | 1.7148 | 1.9998 | 1.7148 | 1.7148 | 1.7148 | 1.9998 |
| Beijing | 0.9999 | 0.9999 | 0.7149 | 1.3849 | 0.9999 | 0.9999 | 0.9999 | 0.9999 | 1.2699 |
| Chongqing | 1.2849 | 1.2849 | 1.2849 | 1.2849 | 1.3849 | 1.2849 | 1.2849 | 0.8848 | 0.8698 |
| Fujian | 0.7149 | 0.7149 | 0.7149 | 0.7149 | 0.7149 | 0.7149 | 0.7149 | 0.7149 | 0.5848 |
| Gansu | 0.5700 | 0.2850 | 0.0000 | 0.0000 | 0.5700 | 0.0000 | 0.0000 | 0.0000 | 0.0000 |
| Guangdong | 1.2849 | 1.2849 | 1.2849 | 1.2849 | 1.4000 | 1.2849 | 1.2849 | 1.2849 | 1.1548 |
| Guangxi | 0.2850 | 0.2850 | 0.2850 | 0.2850 | 0.2850 | 0.2850 | 0.2850 | 0.2850 | 0.2850 |
| Guizhou | 0.2850 | 0.2850 | 0.9999 | 0.4151 | 0.8848 | 0.9999 | 0.9999 | 1.6301 | 1.3451 |
| Hainan | 0.0000 | 0.0000 | 0.0000 | 0.0000 | 0.0000 | 0.0000 | 0.0000 | 0.0000 | 0.0000 |
| Hebei | 1.9998 | 1.9998 | 1.9998 | 1.7148 | 1.3451 | 1.7148 | 1.7148 | 1.9998 | 1.9998 |
| Heilongjiang | 0.0000 | 0.0000 | 0.0000 | 0.0000 | 0.0000 | 0.0000 | 0.0000 | 0.0000 | 0.0000 |
| Henan | 1.9998 | 1.9998 | 1.7148 | 1.7148 | 1.9998 | 1.9998 | 1.9998 | 1.9998 | 1.9998 |
| Hubei | 1.7148 | 1.9998 | 1.9998 | 1.9998 | 1.9998 | 1.9998 | 1.9998 | 1.9998 | 1.9998 |
| Hunan | 1.9998 | 1.9998 | 1.9998 | 1.9998 | 1.9998 | 1.9998 | 1.9998 | 1.9998 | 1.9998 |
| Inner Mongolia | 0.7149 | 0.7149 | 0.9999 | 0.9999 | 0.9999 | 0.9999 | 0.9999 | 0.6547 | 0.8698 |
| Jiangsu | 1.9998 | 1.9998 | 1.9998 | 1.9998 | 1.9998 | 1.9998 | 1.9998 | 1.9998 | 1.7148 |
| Jiangxi | 0.7149 | 1.7148 | 0.7149 | 0.7149 | 0.9999 | 0.9999 | 0.7149 | 1.3696 | 0.9849 |
| Jilin | 0.2850 | 0.2850 | 0.2850 | 0.2850 | 0.0000 | 0.2850 | 0.2850 | 0.0000 | 0.0000 |
| Liaoning | 1.2849 | 1.1849 | 1.1849 | 1.2849 | 0.8999 | 1.0548 | 1.1849 | 0.8001 | 0.5700 |
| Ningxia | 0.3697 | 0.6547 | 0.2850 | 0.3697 | 0.0000 | 0.0000 | 0.0000 | 0.2850 | 0.2850 |
| Qinghai | 0.0000 | 0.0000 | 0.0000 | 0.0000 | 0.0000 | 0.0000 | 0.0000 | 0.0000 | 0.0000 |
| Shananxi | 1.6301 | 1.6301 | 1.6301 | 1.9998 | 1.9998 | 1.9998 | 1.6301 | 1.9998 | 1.9998 |
| Shandong | 1.7148 | 1.9998 | 1.9998 | 1.9998 | 1.7148 | 1.9998 | 1.9998 | 1.9998 | 1.9998 |
| Shanghai | 1.0846 | 0.7149 | 1.0846 | 0.7149 | 1.0846 | 0.7149 | 1.0846 | 0.7149 | 1.0696 |
| Shanxi | 1.1150 | 0.7149 | 1.0999 | 1.0999 | 0.7149 | 0.7149 | 0.9999 | 1.7148 | 1.3451 |
| Sichuan | 0.5151 | 0.5151 | 0.3850 | 0.3850 | 0.2850 | 0.5151 | 0.3850 | 0.8848 | 0.8698 |
| Tianjin | 1.9998 | 1.7148 | 1.9998 | 1.9998 | 1.9998 | 1.9998 | 1.9998 | 0.9999 | 0.8698 |
| Xinjiang | 0.0000 | 0.0000 | 0.0000 | 0.0000 | 0.0000 | 0.0000 | 0.0000 | 0.0000 | 0.0000 |
| Yunnan | 0.0000 | 0.0000 | 0.0000 | 0.0000 | 0.0000 | 0.0000 | 0.0000 | 0.0000 | 0.0000 |
| Zhejiang | 1.7148 | 1.7148 | 1.7148 | 1.7148 | 1.7148 | 1.7148 | 1.7148 | 0.7149 | 1.0696 |

**Appendix C. The MS-AR estimation of the proxy variable Ⅰ.**

| Province | Regime | Parameter | The Proxy Variable Ⅰ | | |
| --- | --- | --- | --- | --- | --- |
|  |  |  | Coefficient | Std. Error | z-Statistic |
| Anhui | Regime 1 | ut(st=1) | 1.157393*** | 0.11558 | 10.01375 |
|  | Regime 2 | ut(st=2) | 1.453296*** | 0.094087 | 15.44625 |
|  |  | | | Regime 1 | Regime 2 |
|  | Constant Transition Probabilities | | Regime 1 | 0.837211 | 0.162789 |
|  |  |  | Regime 2 | 0.116663 | 0.883337 |
|  | Duration | Regime 1 | 6.142937 | Regime 2 | 8.571709 |
| Beijing | Regime | Parameter | The Proxy Variable Ⅰ | | |
|  |  |  | Coefficient | Std. Error | z-Statistic |
|  | Regime 1 | ut(st=1) | 0.696579*** | 0.071488 | 9.743927 |
|  | Regime 2 | ut(st=2) | 0.98592*** | 0.050255 | 19.61821 |
|  |  | | | Regime 1 | Regime 2 |
|  | Constant Transition Probabilities | | Regime 1 | 0.823793 | 0.176207 |
|  |  |  | Regime 2 | 0.114554 | 0.885446 |
|  | Duration | Regime 1 | 5.675128 | Regime 2 | 8.729486 |
| Chongqing | Regime | Parameter | The Proxy Variable Ⅰ | | |
|  |  |  | Coefficient | Std. Error | z-Statistic |
|  | Regime 1 | ut(st=1) | 1.223327*** | 0.049092 | 24.91917 |
|  | Regime 2 | ut(st=2) | 0.997505*** | 0.034092 | 29.25894 |
|  |  | | | Regime 1 | Regime 2 |
|  | Constant Transition Probabilities | | Regime 1 | 0.60986 | 0.39014 |
|  |  |  | Regime 2 | 0.153001 | 0.846999 |
|  | Duration | Regime 1 | 2.563184 | Regime 2 | 6.535899 |
| Fujian | Regime | Parameter | The Proxy Variable Ⅰ | | |
|  |  |  | Coefficient | Std. Error | z-Statistic |
|  | Regime 1 | ut(st=1) | 0.649163*** | 0.020813 | 31.19028 |
|  | Regime 2 | ut(st=2) | 0.884317*** | 0.029434 | 30.04372 |
|  |  | | | Regime 1 | Regime 2 |
|  | Constant Transition Probabilities | | Regime 1 | 0.847597 | 0.152403 |
|  |  |  | Regime 2 | 0.390387 | 0.609613 |
|  | Duration | Regime 1 | 6.561552 | Regime 2 | 2.561563 |
| Gansu | Regime | Parameter | The Proxy Variable Ⅰ | | |
|  |  |  | Coefficient | Std. Error | z-Statistic |
|  | Regime 1 | ut(st=1) | 0.387498*** | 0.040205 | 9.638136 |
|  | Regime 2 | ut(st=2) | 0.599786*** | 0.081569 | 7.353069 |
|  |  | | | Regime 1 | Regime 2 |
|  | Constant Transition Probabilities | | Regime 1 | 0.741958 | 0.258042 |
|  |  |  | Regime 2 | 1 | 4.58E-11 |
|  | Duration | Regime 1 | 3.875334 | Regime 2 | 1 |
| Guangdong | Regime | Parameter | The Proxy Variable Ⅰ | | |
|  |  |  | Coefficient | Std. Error | z-Statistic |
|  | Regime 1 | ut(st=1) | 0.905569*** | 0.038155 | 23.7338 |
|  | Regime 2 | ut(st=2) | 1.03859*** | 0.033352 | 31.14017 |
|  |  | | | Regime 1 | Regime 2 |
|  | Constant Transition Probabilities | | Regime 1 | 0.866942 | 0.133058 |
|  |  |  | Regime 2 | 0.127962 | 0.872038 |
|  | Duration | Regime 1 | 7.515496 | Regime 2 | 7.814832 |
| Guangxi | Regime | Parameter | The Proxy Variable Ⅰ | | |
|  |  |  | Coefficient | Std. Error | z-Statistic |
|  | Regime 1 | ut(st=1) | 0.418364*** | 0.042643 | 9.81086 |
|  | Regime 2 | ut(st=2) | 0.624394*** | 0.043815 | 14.25082 |
|  |  | | | Regime 1 | Regime 2 |
|  | Constant Transition Probabilities | | Regime 1 | 0.875676 | 0.124324 |
|  |  |  | Regime 2 | 0.125634 | 0.874366 |
|  | Duration | Regime 1 | 8.043509 | Regime 2 | 7.95966 |
| Guizhou | Regime | Parameter | The Proxy Variable Ⅰ | | |
|  |  |  | Coefficient | Std. Error | z-Statistic |
|  | Regime 1 | ut(st=1) | 0.570686*** | 0.171789 | 3.322009 |
|  | Regime 2 | ut(st=2) | 1.187924*** | 0.169441 | 7.010843 |
|  |  | | | Regime 1 | Regime 2 |
|  | Constant Transition Probabilities | | Regime 1 | 0.874699 | 0.125301 |
|  |  |  | Regime 2 | 0.129942 | 0.870058 |
|  | Duration | Regime 1 | 7.980809 | Regime 2 | 7.695742 |
| Hainan | Regime | Parameter | The Proxy Variable Ⅰ | | |
|  |  |  | Coefficient | Std. Error | z-Statistic |
|  | Regime 1 | ut(st=1) | 0.218208*** | 0.026031 | 8.38253 |
|  | Regime 2 | ut(st=2) | 0.402697 | 0.038419 | 10.4818 |
|  |  | | | Regime 1 | Regime 2 |
|  | Constant Transition Probabilities | | Regime 1 | 0.891794 | 0.108206 |
|  |  |  | Regime 2 | 0.163531 | 0.836469 |
|  | Duration | Regime 1 | 9.241649 | Regime 2 | 6.115066 |
| Hebei | Regime | Parameter | The Proxy Variable Ⅰ | | |
|  |  |  | Coefficient | Std. Error | z-Statistic |
|  | Regime 1 | ut(st=1) | 1.147046*** | 0.135858 | 8.442983 |
|  | Regime 2 | ut(st=2) | 1.423164*** | 0.059313 | 23.99406 |
|  |  | | | Regime 1 | Regime 2 |
|  | Constant Transition Probabilities | | Regime 1 | 0.390151 | 0.609849 |
|  |  |  | Regime 2 | 0.139033 | 0.860967 |
|  | Duration | Regime 1 | 1.639749 | Regime 2 | 7.192561 |
| Heilongjiang | Regime | Parameter | The Proxy Variable Ⅰ | | |
|  |  |  | Coefficient | Std. Error | z-Statistic |
|  | Regime 1 | ut(st=1) | 0.245068*** | 0.020718 | 11.82877 |
|  | Regime 2 | ut(st=2) | 0.408864*** | 0.023269 | 17.57138 |
|  |  | | | Regime 1 | Regime 2 |
|  | Constant Transition Probabilities | | Regime 1 | 0.882269 | 0.117731 |
|  |  |  | Regime 2 | 0.134506 | 0.865494 |
|  | Duration | Regime 1 | 8.493959 | Regime 2 | 7.434587 |
| Henan | Regime | Parameter | The Proxy Variable Ⅰ | | |
|  |  |  | Coefficient | Std. Error | z-Statistic |
|  | Regime 1 | ut(st=1) | 1.323007*** | 0.057504 | 23.00705 |
|  | Regime 2 | ut(st=2) | 1.568157*** | 0.059863 | 26.19574 |
|  |  | | | Regime 1 | Regime 2 |
|  | Constant Transition Probabilities | | Regime 1 | 0.875915 | 0.124085 |
|  |  |  | Regime 2 | 0.128218 | 0.871782 |
|  | Duration | Regime 1 | 8.058966 | Regime 2 | 7.799226 |
| Hubei | Regime | Parameter | The Proxy Variable Ⅰ | | |
|  |  |  | Coefficient | Std. Error | z-Statistic |
|  | Regime 1 | ut(st=1) | 1.361452*** | 0.053316 | 25.53563 |
|  | Regime 2 | ut(st=2) | 1.547269*** | 0.045331 | 34.13236 |
|  |  | | | Regime 1 | Regime 2 |
|  | Constant Transition Probabilities | | Regime 1 | 0.857783 | 0.142217 |
|  |  |  | Regime 2 | 0.115064 | 0.884936 |
|  | Duration | Regime 1 | 7.031497 | Regime 2 | 8.690811 |
| Hunan | Regime | Parameter | The Proxy Variable Ⅰ | | |
|  |  |  | Coefficient | Std. Error | z-Statistic |
|  | Regime 1 | ut(st=1) | 1.661422*** | 0.031248 | 53.16811 |
|  | Regime 2 | ut(st=2) | 1.431891*** | 0.027722 | 51.65102 |
|  |  | | | Regime 1 | Regime 2 |
|  | Constant Transition Probabilities | | Regime 1 | 0.86559 | 0.13441 |
|  |  |  | Regime 2 | 0.11776 | 0.88224 |
|  | Duration | Regime 1 | 7.439931 | Regime 2 | 8.49182 |
| Inner Mongolia | Regime | Parameter | The Proxy Variable Ⅰ | | |
|  |  |  | Coefficient | Std. Error | z-Statistic |
|  | Regime 1 | ut(st=1) | 0.913277*** | 0.054235 | 16.83917 |
|  | Regime 2 | ut(st=2) | 0.67956*** | 0.1491 | 4.557751 |
|  |  | | | Regime 1 | Regime 2 |
|  | Constant Transition Probabilities | | Regime 1 | 0.892821 | 0.107179 |
|  |  |  | Regime 2 | 0.208285 | 0.791715 |
|  | Duration | Regime 1 | 9.330214 | Regime 2 | 4.801119 |
| Jiangsu | Regime | Parameter | The Proxy Variable Ⅰ | | |
|  |  |  | Coefficient | Std. Error | z-Statistic |
|  | Regime 1 | ut(st=1) | 1.366675*** | 0.049822 | 27.43142 |
|  | Regime 2 | ut(st=2) | 1.55693*** | 0.039458 | 39.45765 |
|  |  | | | Regime 1 | Regime 2 |
|  | Constant Transition Probabilities | | Regime 1 | 0.858439 | 0.141561 |
|  |  |  | Regime 2 | 0.114873 | 0.885127 |
|  | Duration | Regime 1 | 7.064071 | Regime 2 | 8.705297 |
| Jiangxi | Regime | Parameter | The Proxy Variable Ⅰ | | |
|  |  |  | Coefficient | Std. Error | z-Statistic |
|  | Regime 1 | ut(st=1) | 0.760428*** | 0.127867 | 5.947043 |
|  | Regime 2 | ut(st=2) | 1.130938*** | 0.147487 | 7.66804 |
|  |  | | | Regime 1 | Regime 2 |
|  | Constant Transition Probabilities | | Regime 1 | 0.541621 | 0.458379 |
|  |  |  | Regime 2 | 0.724441 | 0.275559 |
|  | Duration | Regime 1 | 2.1816 | Regime 2 | 1.380375 |
| Jilin | Regime | Parameter | The Proxy Variable Ⅰ | | |
|  |  |  | Coefficient | Std. Error | z-Statistic |
|  | Regime 1 | ut(st=1) | 0.596603*** | 0.043788 | 13.62479 |
|  | Regime 2 | ut(st=2) | 0.391929*** | 0.023235 | 16.86794 |
|  |  | | | Regime 1 | Regime 2 |
|  | Constant Transition Probabilities | | Regime 1 | 0.454799 | 0.545201 |
|  |  |  | Regime 2 | 0.137752 | 0.862248 |
|  | Duration | Regime 1 | 1.834186 | Regime 2 | 7.259424 |
| Liaoning | Regime | Parameter | The Proxy Variable Ⅰ | | |
|  |  |  | Coefficient | Std. Error | z-Statistic |
|  | Regime 1 | ut(st=1) | 1.218289*** | 0.101374 | 12.01783 |
|  | Regime 2 | ut(st=2) | 0.929403*** | 0.03565 | 26.06993 |
|  |  | | | Regime 1 | Regime 2 |
|  | Constant Transition Probabilities | | Regime 1 | 4.78E-11 | 1 |
|  |  |  | Regime 2 | 0.128747 | 0.871253 |
|  | Duration | Regime 1 | 1 | Regime 2 | 7.767146 |
| Ningxia | Regime | Parameter | The Proxy Variable Ⅰ | | |
|  |  |  | Coefficient | Std. Error | z-Statistic |
|  | Regime 1 | ut(st=1) | 0.38042*** | 0.047147 | 8.068831 |
|  | Regime 2 | ut(st=2) | 0.563751*** | 0.058345 | 9.662299 |
|  |  | | | Regime 1 | Regime 2 |
|  | Constant Transition Probabilities | | Regime 1 | 0.715778 | 0.284222 |
|  |  |  | Regime 2 | 0.283452 | 0.716548 |
|  | Duration | Regime 1 | 3.518377 | Regime 2 | 3.527934 |
| Qinghai | Regime | Parameter | The Proxy Variable Ⅰ | | |
|  |  |  | Coefficient | Std. Error | z-Statistic |
|  | Regime 1 | ut(st=1) | 0.491154*** | 0.027877 | 17.61832 |
|  | Regime 2 | ut(st=2) | 0.303502*** | 0.024874 | 12.20174 |
|  |  | | | Regime 1 | Regime 2 |
|  | Constant Transition Probabilities | | Regime 1 | 0.865456 | 0.134544 |
|  |  |  | Regime 2 | 0.117703 | 0.882297 |
|  | Duration | Regime 1 | 7.432497 | Regime 2 | 8.495967 |
| Shananxi | Regime | Parameter | The Proxy Variable Ⅰ | | |
|  |  |  | Coefficient | Std. Error | z-Statistic |
|  | Regime 1 | ut(st=1) | 1.522336*** | 0.042367 | 35.93217 |
|  | Regime 2 | ut(st=2) | 1.191977*** | 0.060089 | 19.83679 |
|  |  | | | Regime 1 | Regime 2 |
|  | Constant Transition Probabilities | | Regime 1 | 0.892071 | 0.107929 |
|  |  |  | Regime 2 | 0.165053 | 0.834947 |
|  | Duration | Regime 1 | 9.26532 | Regime 2 | 6.058657 |
| Shandong | Regime | Parameter | The Proxy Variable Ⅰ | | |
|  |  |  | Coefficient | Std. Error | z-Statistic |
|  | Regime 1 | ut(st=1) | 1.330237*** | 0.042378 | 31.38973 |
|  | Regime 2 | ut(st=2) | 1.574992*** | 0.046924 | 33.56501 |
|  |  | | | Regime 1 | Regime 2 |
|  | Constant Transition Probabilities | | Regime 1 | 0.882059 | 0.117941 |
|  |  |  | Regime 2 | 0.135516 | 0.864484 |
|  | Duration | Regime 1 | 8.478849 | Regime 2 | 7.379207 |
| Shanghai | Regime | Parameter | The Proxy Variable Ⅰ | | |
|  |  |  | Coefficient | Std. Error | z-Statistic |
|  | Regime 1 | ut(st=1) | 0.840114*** | 0.042754 | 19.65006 |
|  | Regime 2 | ut(st=2) | 0.609643*** | 0.0478 | 12.75399 |
|  |  | | | Regime 1 | Regime 2 |
|  | Constant Transition Probabilities | | Regime 1 | 1.28E-10 | 1.00E+00 |
|  |  |  | Regime 2 | 1.00E+00 | 5.54E-11 |
|  | Duration | Regime 1 | 1 | Regime 2 | 1 |
| Shanxi | Regime | Parameter | The Proxy Variable Ⅰ | | |
|  |  |  | Coefficient | Std. Error | z-Statistic |
|  | Regime 1 | ut(st=1) | 0.808056*** | 0.070437 | 11.47198 |
|  | Regime 2 | ut(st=2) | 1.214087*** | 0.14502 | 8.37184 |
|  |  | | | Regime 1 | Regime 2 |
|  | Constant Transition Probabilities | | Regime 1 | 0.895266 | 0.104734 |
|  |  |  | Regime 2 | 0.203091 | 0.796909 |
|  | Duration | Regime 1 | 9.54802 | Regime 2 | 4.923911 |
| Sichuan | Regime | Parameter | The Proxy Variable Ⅰ | | |
|  |  |  | Coefficient | Std. Error | z-Statistic |
|  | Regime 1 | ut(st=1) | 0.822291*** | 0.073156 | 11.2402 |
|  | Regime 2 | ut(st=2) | 0.484489*** | 0.068241 | 7.099664 |
|  |  | | | Regime 1 | Regime 2 |
|  | Constant Transition Probabilities | | Regime 1 | 0.857984 | 0.142016 |
|  |  |  | Regime 2 | 0.120401 | 0.879599 |
|  | Duration | Regime 1 | 7.041481 | Regime 2 | 8.305613 |
| Tianjin | Regime | Parameter | The Proxy Variable Ⅰ | | |
|  |  |  | Coefficient | Std. Error | z-Statistic |
|  | Regime 1 | ut(st=1) | 1.201257*** | 0.122881 | 9.775766 |
|  | Regime 2 | ut(st=2) | 1.589012*** | 0.134714 | 11.79548 |
|  |  | | | Regime 1 | Regime 2 |
|  | Constant Transition Probabilities | | Regime 1 | 0.759197 | 0.240803 |
|  |  |  | Regime 2 | 0.314114 | 0.685886 |
|  | Duration | Regime 1 | 4.152773 | Regime 2 | 3.183557 |
| Xinjiang | Regime | Parameter | The Proxy Variable Ⅰ | | |
|  |  |  | Coefficient | Std. Error | z-Statistic |
|  | Regime 1 | ut(st=1) | 0.417916*** | 0.033485 | 12.48058 |
|  | Regime 2 | ut(st=2) | 0.251107*** | 0.034487 | 7.281126 |
|  |  | | | Regime 1 | Regime 2 |
|  | Constant Transition Probabilities | | Regime 1 | 0.875442 | 0.124558 |
|  |  |  | Regime 2 | 0.12588 | 0.87412 |
|  | Duration | Regime 1 | 8.028378 | Regime 2 | 7.94407 |
| Yunnan | Regime | Parameter | The Proxy Variable Ⅰ | | |
|  |  |  | Coefficient | Std. Error | z-Statistic |
|  | Regime 1 | ut(st=1) | 0.472552*** | 0.028925 | 16.33732 |
|  | Regime 2 | ut(st=2) | 0.244937*** | 0.025876 | 9.46575 |
|  |  | | | Regime 1 | Regime 2 |
|  | Constant Transition Probabilities | | Regime 1 | 0.865421 | 0.134579 |
|  |  |  | Regime 2 | 0.117679 | 0.882321 |
|  | Duration | Regime 1 | 7.430553 | Regime 2 | 8.497725 |
| Zhejiang | Regime | Parameter | The Proxy Variable Ⅰ | | |
|  |  |  | Coefficient | Std. Error | z-Statistic |
|  | Regime 1 | ut(st=1) | 1.279334*** | 0.071475 | 17.89902 |
|  | Regime 2 | ut(st=2) | 0.930965*** | 0.147304 | 6.32003 |
|  |  | | | Regime 1 | Regime 2 |
|  | Constant Transition Probabilities | | Regime 1 | 0.881249 | 0.118751 |
|  |  |  | Regime 2 | 0.278032 | 0.721968 |
|  | Duration | Regime 1 | 8.420989 | Regime 2 | 3.596706 |

Regime 1 represents the high-risk state, Regime 2 represents the low-risk state. *** indicates marginal significance at the 1%-level, ** at the 5% -level, and * at the 10% -level. The sample period is 2010~2018. In addition, this paper has tried three regimes and four regimes, but the results show that some standard error of parameters are very large in the three regimes and four regimes. Thus, this paper finally selects the two regimes.

**Appendix D. Sensitivity analysis.**

|  | Average of The Proxy Variable Ⅰ | Average of Individual Risk Index | Average of Contagion Risk Index | Steady-state Probability of Low-risk State | Steady-state Probability of High-risk State | Duration of Low-risk State | Duration of High-risk State | The Proxy Variable Ⅱ | Risk Ranking |
| --- | --- | --- | --- | --- | --- | --- | --- | --- | --- |
| Anhui | 1.2987 | 0.9177 | 1.6797 | 0.8402 | 0.8848 | 6.2584 | 8.6775 | 3.0582 | 7 |
| Beijing | 0.9235 | 0.7922 | 1.0548 | 0.8905 | 0.8323 | 9.1326 | 5.9629 | 2.8445 | 15 |
| Chongqing | 1.1071 | 0.9693 | 1.2450 | 0.5704 | 0.8471 | 2.3276 | 6.5385 | 2.0432 | 22 |
| Fujian | 0.7633 | 0.8680 | 0.6587 | 0.6096 | 0.8476 | 2.5616 | 6.5616 | 1.9195 | 25 |
| Gansu | 0.5902 | 0.9953 | 0.1852 | 0.8597 | 0.4766 | 7.1298 | 1.9107 | 1.7045 | 28 |
| Guangdong | 1.0079 | 0.6746 | 1.3413 | 0.8662 | 0.8811 | 7.4722 | 8.4093 | 3.0526 | 8 |
| Guangxi | 0.6572 | 0.9812 | 0.3333 | 0.8823 | 0.8654 | 8.4941 | 7.4283 | 2.8547 | 14 |
| Guizhou | 0.9092 | 0.9441 | 0.8743 | 0.8657 | 0.8804 | 7.4433 | 8.3620 | 2.9825 | 10 |
| Hainan | 0.3858 | 0.7717 | 0.0000 | 0.8627 | 0.8822 | 7.2825 | 8.4897 | 2.7307 | 19 |
| Hebei | 1.3397 | 0.8679 | 1.8116 | 0.7648 | 0.8960 | 4.2519 | 9.6196 | 2.9450 | 11 |
| Heilongjiang | 0.4722 | 0.9445 | 0.0000 | 0.8809 | 0.8677 | 8.3930 | 7.5602 | 2.7727 | 18 |
| Henan | 1.3937 | 0.8614 | 1.9259 | 0.8750 | 0.8740 | 8.0027 | 7.9335 | 3.2323 | 3 |
| Hubei | 1.4167 | 0.8703 | 1.9630 | 0.8802 | 0.8642 | 8.3445 | 7.3625 | 3.1991 | 5 |
| Hunan | 1.4874 | 0.9748 | 2.0000 | 0.8655 | 0.8824 | 7.4352 | 8.5036 | 3.2860 | 1 |
| Inner Mongolia | 0.9296 | 0.9751 | 0.8842 | 0.8595 | 0.8837 | 7.1173 | 8.5948 | 2.9829 | 9 |
| Jiangsu | 1.4379 | 0.9128 | 1.9630 | 0.8658 | 0.8818 | 7.4494 | 8.4623 | 3.2594 | 2 |
| Jiangxi | 0.9428 | 0.9113 | 0.9743 | 0.4454 | 0.3169 | 1.8030 | 1.4640 | 1.0034 | 29 |
| Jilin | 0.5877 | 0.9532 | 0.2222 | 0.8651 | 0.8821 | 7.4138 | 8.4828 | 2.8400 | 16 |
| Liaoning | 1.0452 | 0.9814 | 1.1090 | 0.8565 | 0.5363 | 6.9688 | 2.1566 | 1.9520 | 24 |
| Ningxia | 0.5652 | 0.8612 | 0.2691 | 0.8953 | 0.7679 | 9.5527 | 4.3080 | 2.4542 | 20 |
| Qinghai | 0.5454 | 1.0908 | 0.0000 | 0.8823 | 0.8655 | 8.4927 | 7.4347 | 2.7967 | 17 |
| Shananxi | 1.3932 | 0.9476 | 1.8388 | 0.8918 | 0.8354 | 9.2429 | 6.0760 | 3.1024 | 6 |
| Shandong | 1.3893 | 0.8527 | 1.9259 | 0.8822 | 0.8653 | 8.4916 | 7.4221 | 3.2166 | 4 |
| Shanghai | 0.7449 | 0.6135 | 0.8763 | 0.0000 | 0.0000 | 1.0000 | 1.0000 | 0.6382 | 30 |
| Shanxi | 0.9361 | 0.8400 | 1.0323 | 0.8921 | 0.8331 | 9.2709 | 5.9933 | 2.8731 | 13 |
| Sichuan | 0.7174 | 0.8946 | 0.5401 | 0.8652 | 0.8822 | 7.4200 | 8.4881 | 2.9074 | 12 |
| Tianjin | 1.3868 | 1.0408 | 1.7327 | 0.8275 | 0.6751 | 5.7983 | 3.0783 | 2.1162 | 21 |
| Xinjiang | 0.4590 | 0.9180 | 0.0000 | 0.6096 | 0.8476 | 2.5614 | 6.5616 | 1.7686 | 26 |
| Yunnan | 0.4668 | 0.9335 | 0.0000 | 0.8492 | 0.6306 | 6.6332 | 2.7069 | 1.7230 | 27 |
| Zhejiang | 1.1672 | 0.8416 | 1.4928 | 0.8494 | 0.5895 | 6.6418 | 2.4359 | 2.0222 | 23 |

**Appendix E. The proxy variable Ⅱ during 2010~2016.**

|  | Average of The Proxy Variable Ⅰ | Average of Individual Risk Index | Average of Contagion Risk Index | Steady-state Probability of Low-risk State | Steady-state Probability of High-risk State | Duration of Low-risk State | Duration of High-risk State | The Proxy Variable Ⅱ | Risk Ranking |
| --- | --- | --- | --- | --- | --- | --- | --- | --- | --- |
| Anhui | 1.2837 | 0.7200 | 1.6798 | 0.8246 | 0.0000 | 5.7012 | 1.0000 | 1.5420 | 23 |
| Beijing | 0.8467 | 0.6084 | 1.0142 | 0.8362 | 0.8039 | 6.1048 | 5.0992 | 2.2619 | 12 |
| Chongqing | 1.1017 | 0.8206 | 1.2992 | 0.8149 | 0.8460 | 5.4029 | 6.4920 | 2.5239 | 7 |
| Fujian | 0.7099 | 0.7028 | 0.7149 | 0.8602 | 0.7407 | 7.1555 | 3.8558 | 2.1099 | 15 |
| Gansu | 0.4342 | 0.7625 | 0.2036 | 0.6336 | 0.0000 | 2.7289 | 1.0000 | 0.7666 | 28 |
| Guangdong | 0.9584 | 0.4704 | 1.3013 | 0.8033 | 0.8495 | 5.0834 | 6.6456 | 2.4569 | 9 |
| Guangxi | 0.4829 | 0.7646 | 0.2850 | 0.8468 | 0.8133 | 6.5281 | 5.3552 | 2.2010 | 13 |
| Guizhou | 0.7244 | 0.7654 | 0.6957 | 0.4123 | 0.5600 | 1.7015 | 2.2730 | 1.0532 | 26 |
| Hainan | 0.2431 | 0.5890 | 0.0000 | 0.8517 | 0.0000 | 6.7418 | 1.0000 | 1.2302 | 24 |
| Hebei | 1.3122 | 0.6406 | 1.7841 | 0.8246 | 0.0000 | 5.7016 | 1.0000 | 1.5568 | 21 |
| Heilongjiang | 0.2913 | 0.7059 | 0.0000 | 0.7416 | 0.8602 | 3.8694 | 7.1519 | 2.0865 | 18 |
| Henan | 1.3968 | 0.6547 | 1.9184 | 0.7819 | 0.8430 | 4.5856 | 6.3697 | 2.5264 | 6 |
| Hubei | 1.4435 | 0.7097 | 1.9591 | 0.8242 | 0.8299 | 5.6881 | 5.8803 | 2.6042 | 4 |
| Hunan | 1.4934 | 0.7727 | 1.9998 | 0.8599 | 0.7461 | 7.1365 | 3.9385 | 2.4615 | 8 |
| Inner Mongolia | 0.8412 | 0.7312 | 0.9185 | 0.8070 | 0.8361 | 5.1825 | 6.1001 | 2.3140 | 11 |
| Jiangsu | 1.4569 | 0.6843 | 1.9998 | 0.8220 | 0.8414 | 5.6166 | 6.3054 | 2.6775 | 1 |
| Jiangxi | 0.8376 | 0.6930 | 0.9392 | 0.7641 | 0.0000 | 4.2384 | 1.0000 | 1.1536 | 25 |
| Jilin | 0.4363 | 0.7094 | 0.2443 | 0.8602 | 0.7407 | 7.1540 | 3.8560 | 1.9905 | 19 |
| Liaoning | 0.9821 | 0.7372 | 1.1542 | 0.8517 | 0.0000 | 6.7413 | 1.0000 | 1.5472 | 22 |
| Ningxia | 0.4188 | 0.6735 | 0.2399 | 0.5345 | 0.0000 | 2.1483 | 1.0000 | 0.6762 | 29 |
| Qinghai | 0.3583 | 0.8682 | 0.0000 | 0.7407 | 0.8603 | 3.8564 | 7.1558 | 2.1098 | 16 |
| Shananxi | 1.3580 | 0.7453 | 1.7885 | 0.8150 | 0.8460 | 5.4040 | 6.4954 | 2.6384 | 3 |
| Shandong | 1.3939 | 0.6475 | 1.9184 | 0.8557 | 0.7259 | 6.9302 | 3.6480 | 2.3408 | 10 |
| Shanghai | 0.7192 | 0.4247 | 0.9262 | 0.0000 | 0.0000 | 1.0000 | 1.0000 | 0.6031 | 30 |
| Shanxi | 0.8172 | 0.6671 | 0.9228 | 0.3958 | 0.2893 | 1.6552 | 1.4071 | 0.8908 | 27 |
| Sichuan | 0.5421 | 0.7066 | 0.4265 | 0.7407 | 0.8602 | 3.8559 | 7.1554 | 2.1943 | 14 |
| Tianjin | 1.4892 | 0.8204 | 1.9591 | 0.8368 | 0.8265 | 6.1284 | 5.7641 | 2.6581 | 2 |
| Xinjiang | 0.3147 | 0.7625 | 0.0000 | 0.8597 | 0.7488 | 7.1278 | 3.9809 | 1.9558 | 20 |
| Yunnan | 0.3095 | 0.7500 | 0.0000 | 0.7406 | 0.8602 | 3.8557 | 7.1554 | 2.0919 | 17 |
| Zhejiang | 1.2794 | 0.6598 | 1.7148 | 0.8086 | 0.8485 | 5.2244 | 6.5993 | 2.6014 | 5 |

**Appendix F. The proxy variable Ⅱ during 2010~2017.**

|  | Average of The Proxy Variable Ⅰ | | Average of Individual Risk Index | | Average of Contagion Risk Index | | Steady-state Probability of Low-risk State | | Steady-state Probability of High-risk State | | Duration of Low-risk State | | Duration of High-risk State | | The Proxy Variable Ⅱ | Risk Ranking | |
| --- | --- | --- | --- | --- | --- | --- | --- | --- | --- | --- | --- | --- | --- | --- | --- | --- | --- |
| Anhui | 1.3084 | 0.7737 | | 1.6842 | | 0.8516 | | 0.0000 | | 6.7405 | | 1.0000 | | 2.0257 | | | 21 |
| Beijing | 0.8586 | 0.6397 | | 1.0124 | | 0.8200 | | 0.8693 | | 5.5559 | | 7.6485 | | 2.8141 | | | 11 |
| Chongqing | 1.0945 | 0.8769 | | 1.2474 | | 0.8146 | | 0.6024 | | 5.3923 | | 2.5154 | | 2.0136 | | | 22 |
| Fujian | 0.7309 | 0.7537 | | 0.7149 | | 0.8735 | | 0.8261 | | 7.9081 | | 5.7501 | | 2.8876 | | | 8 |
| Gansu | 0.4339 | 0.7979 | | 0.1781 | | 0.6938 | | 0.0000 | | 3.2660 | | 1.0000 | | 1.0030 | | | 27 |
| Guangdong | 0.9740 | 0.5112 | | 1.2993 | | 0.8648 | | 0.8460 | | 7.3981 | | 6.4952 | | 3.0222 | | | 7 |
| Guangxi | 0.5051 | 0.8183 | | 0.2850 | | 0.8498 | | 0.8632 | | 6.6597 | | 7.3094 | | 2.8144 | | | 10 |
| Guizhou | 0.8191 | 0.8286 | | 0.8125 | | 0.8360 | | 0.8623 | | 6.0975 | | 7.2613 | | 2.8315 | | | 9 |
| Hainan | 0.2573 | 0.6235 | | 0.0000 | | 0.7783 | | 0.8801 | | 4.5109 | | 8.3419 | | 2.4800 | | | 18 |
| Hebei | 1.3415 | 0.6734 | | 1.8111 | | 0.8420 | | 0.3714 | | 6.3274 | | 1.5909 | | 2.1147 | | | 20 |
| Heilongjiang | 0.3036 | 0.7355 | | 0.0000 | | 0.8267 | | 0.8734 | | 5.7698 | | 7.8988 | | 2.6620 | | | 17 |
| Henan | 1.4263 | 0.7116 | | 1.9286 | | 0.8375 | | 0.8667 | | 6.1556 | | 7.4990 | | 3.1363 | | | 5 |
| Hubei | 1.4562 | 0.7333 | | 1.9642 | | 0.8634 | | 0.8432 | | 7.3223 | | 6.3774 | | 3.1832 | | | 2 |
| Hunan | 1.5191 | 0.8350 | | 1.9998 | | 0.8265 | | 0.8734 | | 5.7637 | | 7.8999 | | 3.1633 | | | 3 |
| Inner Mongolia | 0.8427 | 0.7818 | | 0.8855 | | 0.8617 | | 0.8008 | | 7.2283 | | 5.0211 | | 2.6861 | | | 15 |
| Jiangsu | 1.4736 | 0.7249 | | 1.9998 | | 0.8581 | | 0.8558 | | 7.0451 | | 6.9340 | | 3.2299 | | | 1 |
| Jiangxi | 0.8875 | 0.7374 | | 0.9930 | | 0.0000 | | 0.5277 | | 1.0000 | | 2.1172 | | 0.9510 | | | 28 |
| Jilin | 0.4380 | 0.7572 | | 0.2138 | | 0.8382 | | 0.4510 | | 6.1788 | | 1.8214 | | 1.7582 | | | 26 |
| Liaoning | 0.9818 | 0.7994 | | 1.1099 | | 0.8517 | | 0.0000 | | 6.7415 | | 1.0000 | | 1.8896 | | | 25 |
| Ningxia | 0.4394 | 0.7152 | | 0.2455 | | 0.0000 | | 0.0000 | | 1.0000 | | 1.0000 | | 0.5081 | | | 30 |
| Qinghai | 0.3772 | 0.9140 | | 0.0000 | | 0.8736 | | 0.8262 | | 7.9110 | | 5.7522 | | 2.7374 | | | 12 |
| Shananxi | 1.3923 | 0.7908 | | 1.8150 | | 0.8736 | | 0.8261 | | 7.9087 | | 5.7515 | | 3.1616 | | | 4 |
| Shandong | 1.4207 | 0.6980 | | 1.9286 | | 0.8233 | | 0.8728 | | 5.6579 | | 7.8609 | | 3.0998 | | | 6 |
| Shanghai | 0.7152 | 0.4526 | | 0.8998 | | 0.0000 | | 0.0000 | | 1.0000 | | 1.0000 | | 0.6295 | | | 29 |
| Shanxi | 0.8828 | 0.6851 | | 1.0218 | | 0.0000 | | 0.8713 | | 1.0000 | | 7.7719 | | 1.9069 | | | 24 |
| Sichuan | 0.5965 | 0.7570 | | 0.4838 | | 0.8174 | | 0.8703 | | 5.4773 | | 7.7119 | | 2.6978 | | | 14 |
| Tianjin | 1.4345 | 0.8586 | | 1.8392 | | 0.6643 | | 0.7361 | | 2.9792 | | 3.7898 | | 1.9168 | | | 23 |
| Xinjiang | 0.3305 | 0.8009 | | 0.0000 | | 0.8728 | | 0.8286 | | 7.8645 | | 5.8348 | | 2.7259 | | | 13 |
| Yunnan | 0.3364 | 0.8151 | | 0.0000 | | 0.8261 | | 0.8736 | | 5.7505 | | 7.9086 | | 2.6716 | | | 16 |
| Zhejiang | 1.2219 | 0.6983 | | 1.5898 | | 0.8697 | | 0.0000 | | 7.6764 | | 1.0000 | | 2.1664 | | | 19 |
