# Supplementary material for: Several explorations on how to construct an early warning system for local government debt risk in China
Source: PLoS One. 2022 Feb 8;17(2):e0263391. doi: 10.1371/journal.pone.0263391 (PMC8824348; doi:10.1371/journal.pone.0263391)
Supplement: Attachment — Submitted filename: EVALUATION PAPER _PONE-D-21-31046.pdf [file pone.0263391.s003.pdf]

EVALUATION ÄPER PONE-D-21-31046

1. The paper is too long 37 pages.
2. Question research is clear and intersting
3. Globally, the literature review is rich and up-to-date
4. Tiltles should be improved: too long and sometimes very general such as: algorithm p33
5.
   - **Construction ideas**: this section have to be improved and restructured (page 12)
     - ***Conceptuel framework and theory***
     - ***Litterature review***
     - ***Hypothesis***
   - **Indicator data and methods : Methodology or research design:**

It is preferable to change titles: **Definition** 1,2,3,4,5…. : tiltles must be improved

6. It is not a good idea to insert tables or graphs in the conclusion

Check the references inserted in the text : there are some references which do not appear in the end of the paper

7. Hypothesis are not very clear and not well formulated
8. There is not any appendices but in the text author inserted in page 20; 21;26………
